# A novel small deletion in the *NHS* gene associated with Nance-Horan syndrome

**DOI:** 10.1038/s41598-018-20787-2

**Published:** 2018-02-05

**Authors:** Huajin Li, Lizhu Yang, Zixi Sun, Zhisheng Yuan, Shijing Wu, Ruifang Sui

**Affiliations:** Department of Ophthalmology, Peking Union Medical College Hospital, Peking Union Medical College, Chinese Academy of Medical Sciences, Beijing, 100730 China

## Abstract

Nance-Horan syndrome is a rare X-linked recessive inherited disease with clinical features including severe bilateral congenital cataracts, characteristic facial and dental abnormalities. Data from Chinese Nance-Horan syndrome patients are limited. We assessed the clinical manifestations of a Chinese Nance-Horan syndrome pedigree and identified the genetic defect. Genetic analysis showed that 3 affected males carried a novel small deletion in *NHS* gene, c.263_266delCGTC (p.Ala89TrpfsTer106), and 2 female carriers were heterozygous for the same variant. All 3 affected males presented with typical Nance-Horan syndrome features. One female carrier displayed lens opacities centered on the posterior Y-suture in both eyes, as well as mild dental abnormalities. We recorded the clinical features of a Chinese Nance-Horan syndrome family and broadened the spectrum of mutations in the *NHS* gene.

## Introduction

Nance-Horan syndrome (NHS)[MIM 202350], characterized by congenital cataracts, distinctive dental and facial abnormalities, is a rare X-linked recessive inherited disease first described by Nance and Horan in 1974^1,2^.While its prevalence is still unknown, to date, Nance-Horan syndrome has been reported in Caucasian, Turkish, Tunisian, Arabian, Indian and Chinese ethnic groups^1–9^. Affected males have severe bilateral congenital dense cataracts, distinctive dental and craniofacial abnormalities^10^. Microcornea, microphthalmia, hand and foot malformations and mild to moderate intellectual disability have also been recorded in some families^11,12^. Female carriers have lens opacities involving posterior Y-suture, with milder extra-ocular manifestations^5^.

The *NHS* gene, located on Xp22.13, has been linked with this rare disorder^13^. *NHS* is abundantly expressed during the development of embryonic tissues, particularly in lens, brain, craniofacial mesenchyme, and primordial teeth^10^. At least 4 isoforms can result from alternative splicing. Isoform A (NHS-A), the major isoform, encodes a 1630-amino acid protein which is located in the epithelial cell membrane and may interact with the tight junction protein zona occludens-1(ZO-1)^14,15^. A further study indicated that the NHS protein is a novel regulator of actin remodeling and cell morphology^16^. The actual function, regulation and interaction of NHS proteins remain not fully understood. About 40 causative mutations in *NHS* have been reported (HGMD Database; http://www.hgmd.cf.ac.uk), most of which are nonsense mutations or small deletions.

Though approximately 60 Nance-Horan syndrome families or cases have been identified worldwide^1,3–8,10,12,17–29^, only 4 were Chinese^4,9,12,18^. This may be due to the lack of awareness of this rare syndrome. In the present study, we characterized the clinical features of a Chinese pedigree with Nance-Horan syndrome and a novel molecular variant in *NHS*.

## Results

### Clinical evaluation

**Figure 1:**
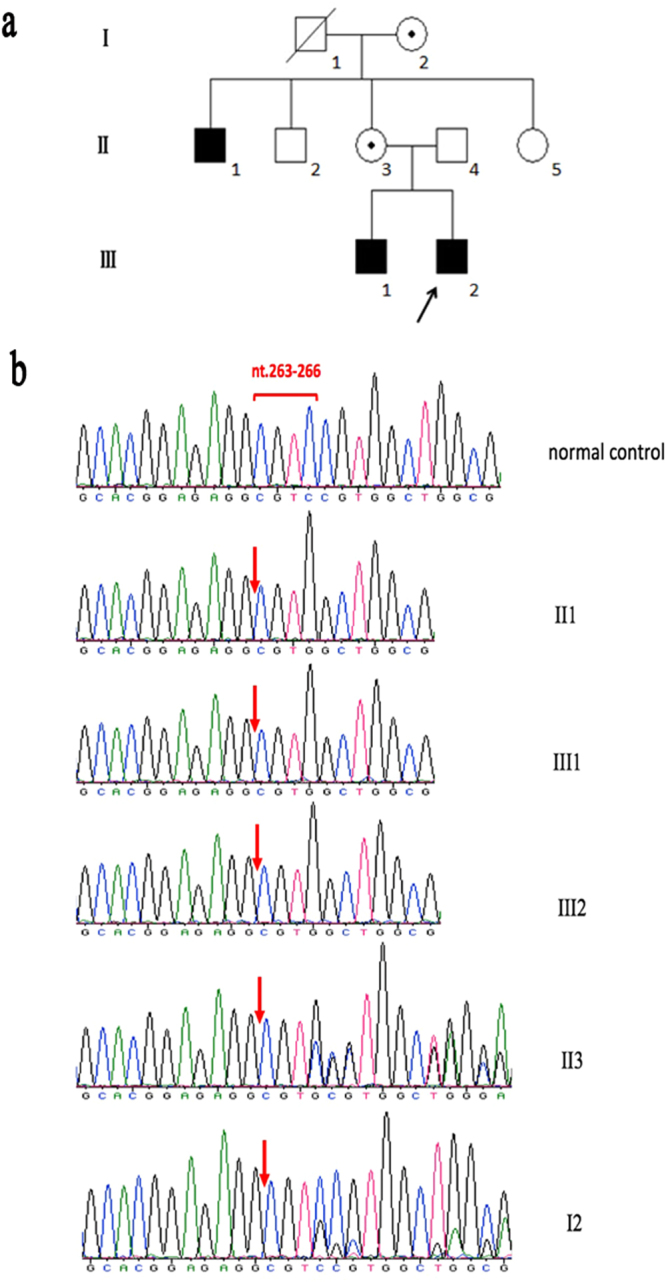
The pedigree and sequencing results of the Nance-Horan syndrome family. (**a**) The pedigree exhibited an X-linked recessive inheritance mode. Solid symbols indicate affected individuals; open symbols indicate normal subjects; symbols with a dot inside indicate mutant allele carriers; slashed symbols indicate deceased individuals; a square represents a male and a circle, a female individual. An arrow marks the proband. (**b**) The sequencing results show a 4-base-pair deletion (CGTC) at nucleotide 263 causing a frameshift in codon 89 and a premature termination of translation (p.Ala89TrpfsTer106). The female carriers were heterozygous for the same mutation. nt. refers to nucleotide.

**Figure 2:**
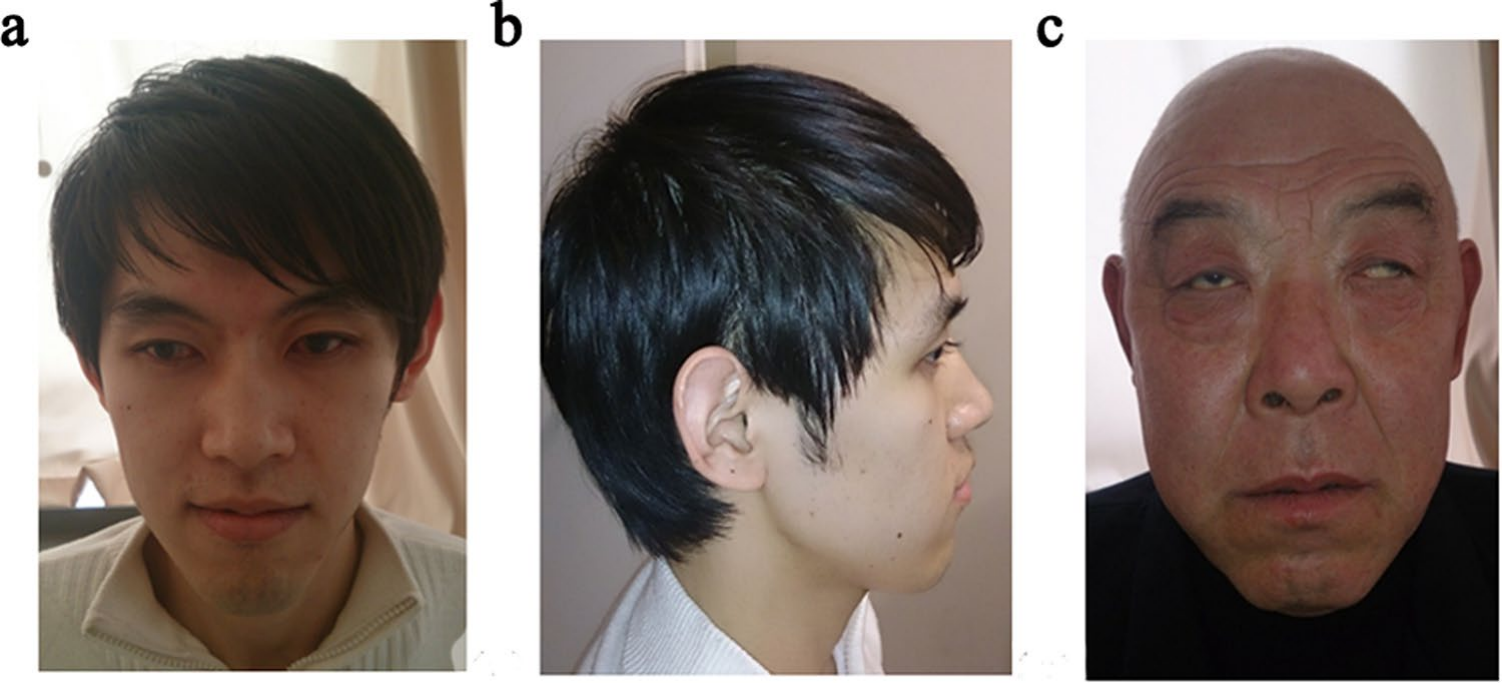
Representative facial dysmorphology of the NHS patients. (**a**) The frontal view of III2. III2 had a long and narrow face. (**b**) The lateral view of III2. III2 had large, anteverted and mild enlarged pinnae. (**c**)Frontal view of II1. II1 had a bulbous nose.

**Figure 3:**
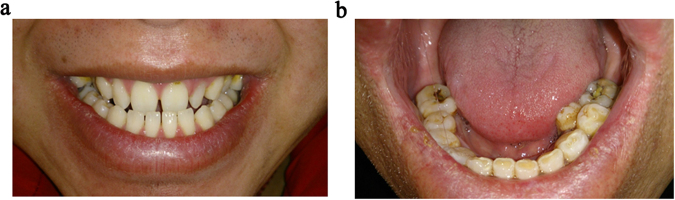
Representative dental abnormalities of the NHS patients. (**a**) Screw-driver like incisors of III1. (**b**) Mulberry-like molars, crowded premolars and missing of the second molars of II1.

The proband, his brother and one of his uncles were affected while the female members of the family were unaffected. The pedigree suggested an X-linked recessive pattern of inheritance (Fig. 1). All the affected males had poor vision since birth. They were diagnosed with bilateral congenital cataracts and cataract surgery was performed at an early age. Seven years later, the affected brothers (III1 and III2) developed bilateral glaucoma. Although they underwent glaucoma surgeries and medical treatment, intraocular pressure (IOP) was poorly controlled, being around 50 mmHg. II1 complained of intermittent swelling pain of the left eye when he was around age 20, but he had never visited a doctor. When the affected males were referred to our clinic, the best corrected visual acuity (BCVA) ranged from no light perception (NLP) to 0.05. Bilateral cornea cloudy opacity with the appearance of calcium in central cornea(band keratopathy) was present in III1 and III2. Their IOPs were unmeasurably high (>50 mmHg). The IOP of II1 was within normal limits. He had band keratopathy in the left eye. Microcornea (cornea diameter = 9 mm), exotropia and nystagmus were present in all the patients. They all had a long-narrow face (Fig. 2a), large anteverted and mild enlarged pinnae (Fig. 2b). II1 displayed a bulbous nose (Fig. 2c). Dental abnormalities were identified in all individuals, and included screw-driver like incisors (Fig. 3a), mulberry-like molars, and various examples of dental agenesis (Fig. 3b).

**Figure 4:**
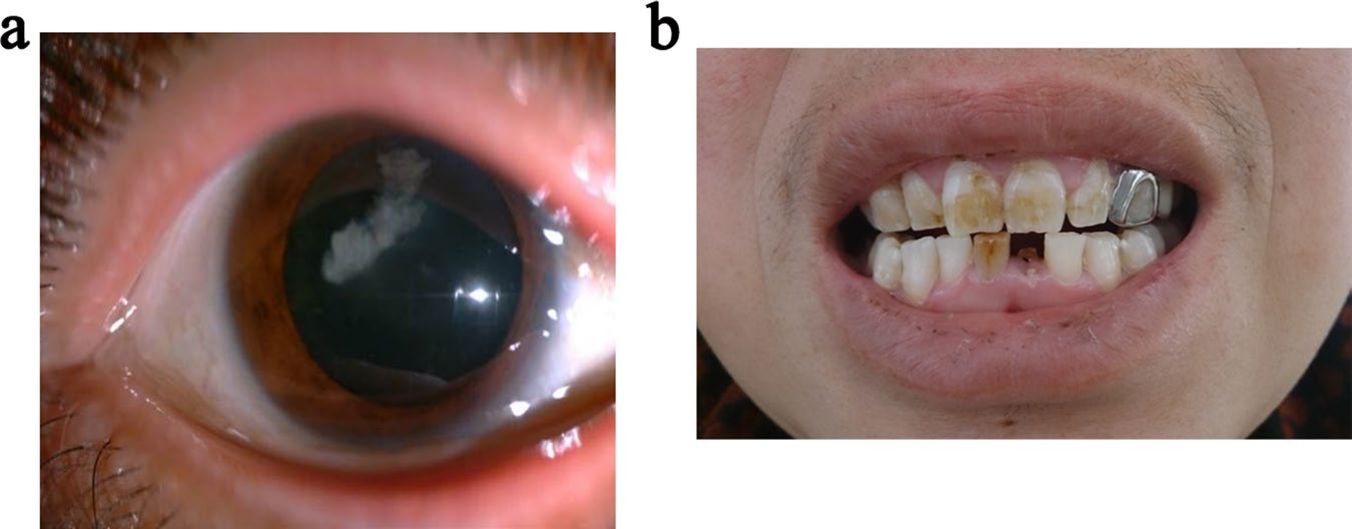
Phenotypes of the female carrier (II3). (**a**) Slit-lamp photograph of II3. Lens opacities centered on the posterior suture, both eyes. (**b**) Dental abnormalities of II3. Her teeth exhibited mild screw-driver shape incisors and mulberry-like molars. Her left central incisor appeared “normal” according to her description and was subsequently broken accidentally.

The proband’s mother(II3) was a 50-year-old female with no history of ocular disease except for high myopia. The cornea diameter of both eyes was 12 mm. Her BCVA was 0.15 OD and 0.4 OS. Slit-lamp examination of the both eyes revealed lens opacities centered on the posterior Y-suture (Fig. 4a). She had a normal facial gestalt and ears. Her incisors exhibited mild screw-driver shape and mulberry-like molars. Her left central incisor appeared “normal” according to her description and was subsequently broken accidentally (Fig. 4b). The proband’s grandmother (I2) was unavailable for examination. According to her medical history, she was diagnosed as bilateral cataracts at the age of 58, and had already received surgical treatment.

No hand and foot abnormality, or intellectual disability were observed among all our subjects. Detailed clinical evaluation of the affected males and the female carriers is summarized in Table 1.

**Table 1:** Clinical features of the affected males and the female carriers.

	Affected male patients			Female carriers	
	II1	III1	III2	II3	I2
**Age/gender**	54/M	24/M	22/M	50/F	80/F
**BCVA**	0.05/LP	NLP/NLP	LP/LP	0.15/0.4	NA
**Ocular features**					
Congenital cataracts	**+**	**+**	**+**	Y suture opacity	**+**
Cataract surgery	**+**	**+**	**+**	−	**+**
Glaucoma	**+**	**+**	**+**	−	NA
Strabismus	**+**	**+**	**+**	−	NA
Nystagmus	**+**	**+**	**+**	−	NA
Others	band keratopathy	cornea cloudy opacity, band keratopathy	cornea cloudy opacity, band keratopathy	high myopia	NA
**Facial abnormalities**					
Long-narrow face	**+**	**+**	**+**	−	NA
Bulbous nose	**+**	−	−	−	NA
Anteverted and mild enlarged pinnae	**+**	**+**	**+**	−	NA
**Dental abnormalities**					
Screw-driver shaped incisors	**+**	**+**	**+**	**+**	NA
Mulberry-like molars	**+**	**+**	**+**	**+**	NA
Dental agenesis	missing of the second molars	−	crowded premolars, and missing of the second molars	broken left central incisor	NA
**Hand and foot abnormalities**	−	−	−	−	NA
**Intellectual disability**	−	−	−	−	NA

BCVA, best corrected visual acuity; LP, light perception; NLP, no light perception; NA, not available.

### Genetic analysis

We identified a novel disease-causing *NHS* mutation in the family. The sequencing results showed a 4-base-pair deletion (CGTC) at nucleotide 263–266 in exon 1 of *NHS* gene, which would cause a frameshift from codon 89 and a premature termination of translation. (p.Ala89TrpfsTer106). The variant sequence was confirmed by segregation in the pedigree (Fig. 1).

## Discussion

Congenital cataract is the leading cause of irreversible blindness in childhood^30^. Approximately half of congenital cataracts are inherited, either with and without other ocular anomalies or as part of multisystem genetic disorders. Inherited cataracts are most frequently inherited as autosomal dominant traits, but also could be inherited in an autosomal recessive or X-linked pattern^31^. Nance-Horan syndrome is one of the few syndromes with cataract that is inherited as an X-linked trait. In contrast to other well-studied subgroups of genes associated with congenital cataract such as the crystallins^32^, *NHS* was speculated to play a vital role in cell-to-cell tight conjunction formation together with the tight junction protein, ZO-1^15^. NHS-A protein was normally localized at cellular periphery of various tissues, especially lens epithelium^16,25,33^. Mutant NHS-A protein was found in cytoplasm^25^. Normally functioned intercellular junctions are important in lens development and maintaining lens homeostasis^34,35^. The dislocation of the NHS protein and altered intercellular contacts are likely to underlie cataract formation in Nance–Horan syndrome. A novel small deletion (c.263_266delCGTC) was identified in our study that would result in a truncated protein (p.Ala89TrpfsTer106). Nonsense mutations and small deletions are the most common mutations in *NHS*^3^. Small insertions, large deletions, large insertions, splice site mutations and missense mutations have also been reported (HGMD Database; http://www.hgmd.cf.ac.uk). Currently, only 4 mutations in Chinese patients with Nance-Horan syndrome have been identified, including a nonsense mutation (p.E108X), a small deletion (c.852delG), splice site mutation (c.1045 + 2 T > A) and a large deletion at Xp22.13^4,9,12,18^.

Congenital cataract is the most prominent feature of Nance-Horan syndrome and leads to profound vision loss and greatly affects the quality of life. The three affected males in our study had congenital bilateral dense nuclear cataracts and received cataract surgery at an early age. The BCVA of our NHS patients ranged from NLP to 0.05. Almost all NHS patients undergo cataract extraction but the overall prognosis for vision is still poor, mostly NLP to 0.3^5,8,9,18,24^. Delayed surgical intervention, amblyopia and glaucoma may contribute to the poor visual outcomes. All affected males in our study developed glaucoma.According to previous reports, about 10% of NHS patients exhibited secondary glaucoma^5,7,26^. This number might be underestimated because some NHS patients were first diagnosed by dentists or they did not visit doctor to check IOP, like II1. Poorly developed anterior chamber angles in NHS was reported^26^, which was suggested that the abnormal aqueous humor drainage system contribute to recurrent secondary glaucoma. None of the other reported Chinese NHS patients exhibited glaucoma. In addition to congenital cataracts and glaucoma, our patients also manifested other common ocular features of NHS, including microcornea, strabismus and nystagmus.

Multi-systemic abnormalities in Nance-Horan syndrome are easily overlooked by ophthalmologists, which may lead to inaccurate diagnosis. The affected males in the present study displayed long-narrow face, anteverted and mild enlarged pinnae. II1 exhibited a bulbous nose. All of them presented screw-driver shaped incisors, mulberry-like molars and various dental agenesis including crowded premolars and missing second molars. Long-narrow face, anteverted and mild enlarged pinnae and bulbous nose are the characteristic facial features of NHS. The severity of these phenotypes varied from case to case^3,6,8,12,18^. Screw-driver shaped incisors and mulberry-like molars are the most distinctive dental abnormalities of NHS^24^. A spectrum of other dental abnormalities, such as diastema, supernumenary teeth, and dental agenesis were commonly observed^24^. Spontaneous dental abscess was recorded in one case^36^. None of our patients exhibited hand and foot abnormalities, and all had normal intellect. Most common hand and foot abnormalities are broad or short fingers and brachymetacarpalia^5,12^. Sandal gap and partial syndactyly of toes were reported in one Turkish NHS family^3^. About 30% of affected males have mild to moderate intellectual disability^11^. Three of the four reported Chinese cases manifest typical NHS features as in the present study, including congenital cataracts, distinctive dental and craniofacial abnormalities^12,18,37^. The NHS patients in Liao’s study also displayed hand and foot abnormalities, psychomotor retardation, and cryptorchidism, probably as the microdeletion encompasses the *REPS2*, *NHS*, *SCML1* and *RAI2* genes^4^. The cytogenetic abnormalities involving the flanking genes in Xp22.13 may contribute to the variability of phenotypes such as cryptorchidism and tetralogy of Fallot^4,21^. However, intellectual disability can present in patients with only *NHS* gene mutation, which indicated that *NHS* play a vital role in mental development. Three of the four reported Chinese cases were diagnosed by next generation sequencing, systemic manifestations were not stated in two of them^9,12,18^. These reports indicate that this rare disorder can be unrecognized, especially when the phenotype is not typical. When checking patients with congenital cataracts, a comprehensive medical history is vital to determine if other organs are involved. A basic assessment of facial, dental, skeletal, genitourinary and neurological abnormalities are important for accurate diagnosis.

Like many other X-linked inherited ocular diseases, such as X-linked retinitis pigmentosa and choroideremia^37,38^, female carriers of Nance-Horan syndrome can have variable mild phenotypes. It’s mainly due to skewed X inactivation^39^. II3 experienced declined vision, with a BCVA of 0.15 OD and 0.4 OS. Female carriers of NHS may remain normal vision^5,18^. II3 displayed bilateral lens opacities centered on the posterior Y-suture. I2 developed bilateral cataracts that required surgery in her late 50′s. It is speculated that the cataracts in I2 were more severe than II3. Y-suture opacity is considered to be a sensitive and specific clinical sign for female carriers of Nance-Horan syndrome^5^. Nuclear opacity or clear lens was also reported in a small number of cases^3,4,18^. Besides the typical Y-suture opacity feature, II3 exhibited mild dental abnormalities including screw-driver shape incisors and mulberry-like molars. Heterozygous females of Nance-Horan syndrome may often manifest similar but less pronounced extraocular features than affected males^6,29^.

In conclusion, our study identifies a novel pathogenic *NHS* gene mutation in a Chinese pedigree clinically diagnosed with Nance-Horan syndrome. Our findings broaden the spectrum of mutations associated with Nance-Horan syndrome in Chinese population, and shed light on the diagnosis of this rare disease.

## Methods

### Clinical evaluation

Two male patients with congenital cataracts were identified at the Ophthalmic Genetic Clinic at Peking Union Medical College Hospital (PUMCH), Beijing, China. After checking their uncle and mother, X-linked recessive inheritance was suspected. Detailed medical and family histories were taken. Three affected males and 1 female carrier underwent ophthalmic evaluations, including BCVA according to the decimal Snellen E chart, intraocular pressure (IOP), cornea diameter measurement and slit-lamp biomicroscopy. Non-ocular features were documented including the morphology of teeth, face, ears, hands and feet. The study protocol was approved by the Institutional Review Board of PUMCH and adhered to the tenets of the Declaration of Helsinki. Informed consent was obtained from all subjects for both study participation and publication of identifying images.

### Genetic analysis

Genomic DNA was isolated from peripheral blood with the QIAamp DNA Blood Midi Kit (Qiagen, Hilden, Germany) according to the manufacturer’s protocol. Polymerase chain reactions (PCR) were designed to amplify the *NHS* exons and splice-site sequences. Primers were synthesized according to sequences published previously^25^. For exon 2 to 8, the final volume of 50 μl contained 40ng DNA, 10pmol of each primer, and 25 μl 2× Taq PCR Master Mix (Biomed Technologies, Beijing, China). For exon1, the final volume of 50 μl contained 2 × PCR buffer, 5× Q solution, dNTP mix(10 mM of each),10pmol of each primer, deionized and distilled water, and HotStarTaq DNA Polymerase (Qiagen, Hilden, Germany). The amplification was performed under the following conditions: 95 °C for 5 min, followed by 33 cycles at 95 °C for 30 s, 60 °C for 30 s, 72 °C for 45 s, with a final extension at 72 °C for 7 min. The PCR products were purified using *EasyPure* PCR purification Kit (Transgen Biotech, Beijing, China). The amplicons were sequenced using forward and reverse primers on an ABI 3730 Genetic Analyzer (ABI, Foster City, CA). The sequences were assembled and analyzed using Lasergene SeqMan software (DNASTAR, Madison, WI). All available family members were Sanger sequenced in order to confirm segregation of the mutation.
